# Modeling a mesenchymal cell state by bioprinting for the molecular analysis of dormancy in melanoma

**DOI:** 10.1016/j.mtbio.2025.101674

**Published:** 2025-03-18

**Authors:** Sonja K. Schmidt, Stefan Fischer, Zubeir El Ahmad, Rafael Schmid, Eric Metzger, Roland Schüle, Claus Hellerbrand, Andreas Arkudas, Annika Kengelbach-Weigand, Melanie Kappelmann-Fenzl, Anja K. Bosserhoff

**Affiliations:** a Institute of Biochemistry, Friedrich-Alexander-University Erlangen-Nürnberg (FAU), Fahrstraße 17, 91054, Erlangen, Germany; b Faculty of Computer Science, Deggendorf Institute of Technology, Dieter-Görlitz-Platz 1, 94469, Deggendorf, Germany; c Laboratory for Tissue-Engineering and Regenerative Medicine, Department of Plastic and Hand Surgery, University Hospital Erlangen, Friedrich-Alexander-University Erlangen-Nürnberg (FAU), 91054, Erlangen, Germany; d Klinik für Urologie und Zentrale Klinische Forschung, Universitätsklinikum Freiburg, Medizinische Fakultät, Albert-Ludwigs-University Freiburg, 79106, Freiburg, Germany; e CCC Erlangen-EMN: Comprehensive Cancer Center Erlangen-EMN (CCC ER-EMN), 91054, Erlangen, Germany; f CCC WERA: Comprehensive Cancer Center Alliance WERA (CCC WERA), 91054, Erlangen, Germany; g BZKF: Bavarian Cancer Research Center (BZKF), 91054, Erlangen, Germany

**Keywords:** Cancer, 3D cell culture, Bioinformatics, Quiescence, Mechanosensation

## Abstract

Malignant melanoma is a highly aggressive tumor originating from the pigment producing cells, the melanocytes. It accounts for the majority of skin cancer related deaths worldwide. This is often due to the development of therapy resistance or tumor dormancy, eventually resulting in tumor relapse by yet undefined mechanisms. Tumor dormancy is thought to be mediated by the cellular microenvironment and models taking this factor into account are urgently needed.

We 3D bioprinted melanoma cells in the hydrogels Cellink Bioink (CIB) or Matrigel (MG), each as a substitute of the extracellular matrix, and, thereby, induced a quiescent or a proliferative phenotype of the melanoma cell lines, respectively. RNA-Seq with subsequent comprehensive bioinformatical and molecular analyses assigned CIB-cultured cells to a predominantly mesenchymal and Matrigel-cultured cells to a more mitotic phenotype, emphasizing the CIB model as a suitable platform for the investigation of dormancy under consideration of the microenvironment. Melanoma cells in CIB 3D culture reflect a quiescent and migratory active cell state e.g. by revealing significant downregulation of genes associated with replication and cell cycle progression in this setting. Using this model system, we identified the mechanosensory gene FHL2 as one early sensor of changes in the ECM and suggest a FHL2-p21/AP-1 axis contributing to the dormant phenotype of melanoma cells in CIB.

## Graphical abstract

**Figure:**
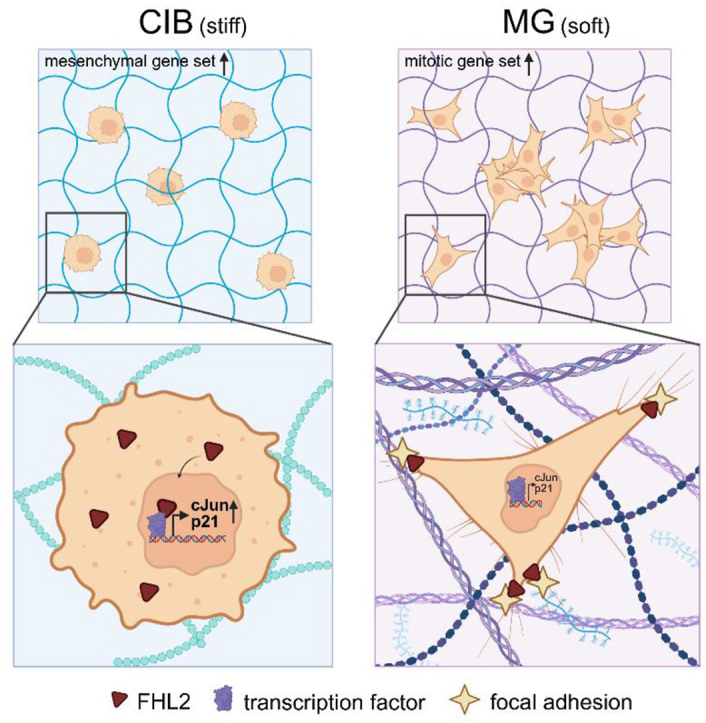

## 1 Introduction

Late recurrence is an issue in many cancers including malignant melanoma. Studies suggest that these recurrences may be caused by undetected former sleeping tumor cells within the patient, which awake and lead to aggressive metastases. The mechanisms coordinating this so-called *tumor dormancy*, as well as their awakening are barely understood.

It is known that epithelial to mesenchymal transition (EMT) promotes motility and invasiveness of tumor cells, thus enabling dissemination from the primary tumor. In the context of tumor dormancy, Weidenfeld and Barkan nicely summarized that mesenchymal-like disseminated tumor cells (DTCs) may stay dormant after colonizing the distant organ, whereas metastatic growth may be dependent on DTCs ability to regain their epithelial phenotype by mesenchymal to epithelial transition (MET) [1]. Until today, various studies have shown a link between epithelial-mesenchymal transition (EMT), stemness, and the metastatic initiating potential of DTCs. It is known that only a very small percentage of cancer cells, which disseminate from the primary tumor, survive this process and can persist at distant sites [2]. Accumulating evidence suggests that, within a heterogeneous tumor, only the subpopulation of so-called cancer stem cells (CSCs) might be responsible for the tumor's ability to develop, invade and disseminate into distant organs. CSCs possess the ability of self-renewal, differentiation and resistance to cancer therapy by inducing a quiescent state. CSCs are characterized by their high plasticity in response to external stimuli and therefore, are substantially influenced by their tumor microenvironment or specific niche. Dormant tumor cells share some stem-like characteristics, including quiescence and plasticity. However, they will shift to self-renewal and with this to proliferation upon respective cues from their niche. Clearly, these processes require high plasticity of the DTCs, indicating the complexity of the molecular characterization of these cells.

Given the impact of the tumor niche on the cellular phenotype, classic 2D cell culture is not well suited for the investigation of the above-mentioned phenomena. 3D cultivation methods enable a more physiological resemblance of the cell's microenvironment in terms of cell-cell and cell-matrix interactions, as well as nutrient and oxygen gradients. The field of biofabrication is about to take 3D cell culture even further. Using techniques like 3D bioprinting, cells are incorporated within a specified, printable hydrogel and deposited with unprecedented spatial control.

Previous studies revealed that biofabrication offers the possibility to mimic defined situations for tumor cells in 3D [[3], [4], [5]]. Further, the two materials, Cellink Bioink (CIB) and Matrigel (MG), were characterized in depth concerning hydrogel characteristics and cell behavior [6,7]. MG is a basement membrane like matrix composed of type IV collagen, laminin, entactin, heparan sulfate, proteoglycans, and various growth factors thereby offering a typical pathophysiological tumor microenvironment. CIB on the contrary is an artificial matrix composed of alginate and cellulose, which mammalian cells do not face *in vivo*.

Our previous publications show that melanoma cells printed in MG can proliferate, while they develop a non-proliferative (G1 cell cycle arrest) but migratory phenotype in the bioprinted hydrogel CIB *in vitro* and *in vivo* using CIB as a matrix in the arteriovenous (AV) loop model in immunodeficient rats. We suggest that not the composition, but mechanical or topological properties of CIB deliver the crucial signals for the cells to become dormant. In general, we did not use the two fundamentally different hydrogels to directly mimic specific tissues, but rather to mimic the phenotypes of interest, namely the proliferation in MG, and dormancy in CIB. Besides other differences discussed in a previous study [7], CIB and MG differ significantly in their degradability, presentation of adhesion peptides, as well as in their stiffness, showing in Young's moduli of 67.9 ± 7.3 kPa for CIB and 43 ± 10.6 kPa for MG.

With the present publication, we now shed light on the molecular mechanisms underlying the observed dormant cell behavior by RNA-Seq and validate the bioprinted CIB culture as a highly potent model for the in-depth analysis of the dormant phenotype.

## 2 Materials and methods

### 2.1 Cell culture

Human melanoma cell lines Mel Im (FUCCI) and MV3 (FUCCI) were cultured and used as described previously [8,9]. Briefly, Mel Im (FUCCI) were cultivated in DMEM low glucose and MV3 (FUCCI) in DMEM high glucose, each supplemented with 10 % FCS and 1 % Pen/Strep at 37 °C and 8 % CO_2_ in humidified atmosphere. Mycoplasma contamination was regularly excluded for all cell lines. Parental cell lines were authenticated using the short tandem repeat (STR) method.

### 2.2 3D bioprinting

Bioprinting was performed as described before in detail [3]. Briefly, cells were harvested, and suspensions were mixed 1:11 with Cellink Bioink (CIB) (#IKC20000; BICO Group, Gothenburg, Sweden) or ice-cold Matrigel (MG) (#354234, Corning Inc., Corning, NY, USA) to a final concentration of 3 × 10^5^ cells/ml, respectively. The MG bioink (final concentration: 8.2 mg/ml – 9.5 mg/ml) was pre-gelled at room temperature for 30 min. For both materials, three-layered grid structures (1 cm^2^) were printed into 6-well plates. CIB constructs were crosslinked with 50 mM CaCl_2_, for 5 min, whereas MG constructs were polymerized by a temperature shift to 37 °C, for 30 min. Hardened constructs were cultivated in the respective cell culture medium at 37 °C and 8 % CO_2_.

### 2.3 siRNA transfection

The melanoma cell lines Mel Im or MV3 were transfected with siRNA pools for CDKN1A (p21), KLF4 or FHL2, and a control pool (siCtrl), respectively (siTOOLs Biotech, Planegg, Germany) using the Lipofectamine RNAiMAX reagent (Thermo Fisher Scientific, Waltham, MA, USA). For knockdown analysis 200,000, 160,000 or 120,000 cells were seeded into the wells of a 6-well plate, and transfected for 24 h, 48 h or 72 h, respectively, before harvest. For 3D experiments, cells were transfected 24h before printing into CIB.

### 2.4 qRT-PCR

RNA from Mel Im cell pellets for knockdown analysis was extracted using the E.Z.N.A. Total RNA Kit I (Omega Bio-tek, Inc., Norcross, GA, USA). RNA from bioprinted constructs was extracted after two days in culture using Trizol® Reagent (Thermo Fisher Scientific, Waltham, MA, USA) as described elsewhere [8,10]. The resulting RNA-pellets were resuspended in nuclease-free water. RNA from cell pellets was measured using the Nanodrop 2000 and 500 ng were used for reverse transcription into cDNA using SuperScript II Reverse Transcriptase (Thermo Fisher Scientific, Waltham, MA, USA) as described before [11]. As the yields of CIB samples were too low for valid nanodrop measurements due to low cell numbers, 4 μl of the CIB RNA and 10 ng of MG RNA were reversely transcribed following the same method.

For real-time PCR, the LightCycler® 480 II (Roche, Basel, Switzerland) was used with primers from Sigma-Aldrich (St. Louis, MO, USA) (Suppl. Table 1).

### 2.5 Western Blot

Western Blot protein analysis of Mel Im with FHL2 knockdown versus control treatment was performed using techniques described previously [12]. Immunoblots were probed using FHL2 primary antibody (1:1000, HPA006028, Sigma Aldrich, St. Louis, MO, USA) in 5 % milk powder in TBS-T at 4 °C overnight. Anti-β-actin antibody staining (1:5000, #A5441, Sigma Aldrich, St. Louis, MO, USA) was used as loading control. After washing the respective antibodies were labelled using a horseradish peroxidase-coupled secondary antibody (1:2000, anti-rabbit HRP, #7074, or anti-mouse-HRP, #7076, Cell Signaling, Danvers, MA, USA) in TBS-T. Immunoreactions were visualized using an ECL staining kit (Bio-Rad, Hercules, CA, USA) and the ChemoStar Blot Imager (Intas, Göttingen, Germany). Quantification of band intensities was performed using the Labimager Software (Kapelan, Leipzig, Germany).

### 2.6 Immunohistochemistry

For immunohistochemical analysis of Mel Im in CIB or MG culture, constructs were printed as described and harvested after 2 days in culture. The CIB-gels were fixed in 4 % PFA, MG in 2 % PFA for 15 min. Fixed samples were dehydrated in an alcohol series and embedded in paraffin before cutting 5 μm slices for subsequent staining. Immunohistochemical staining was performed as described previously, using primary antibodies anti-p21 (1:50, #2947, Cell Signaling, Danvers, MA, USA) or anti-KLF4 (1:500, HPA002926, Sigma Aldrich, St. Louis, MO, USA) [13]. P21-staining on CIB- and MG-sections was described quantitatively as “none”, “weak”, “moderate” or “strong”. Therefore, at least 70 cells were counted on three independent sections per condition.

### 2.7 Immunofluorescence

Immunofluorescent staining of cells in CIB and MG was performed on 5 μm paraffin sections, prepared as described above. Briefly, antigen retrieval was carried out in citrate-buffer pH 6.0 with 0.5 % Tween-20 for 40 min at 95 °C before sections were permeabilized with 1 % Triton X-100 in PBS and blocked with 5 % BSA/PBS. Antibodies against c-Jun (1:50, sc-166540, Santa Cruz, Dallas, TX, USA), FHL2 (1:200, HPA006028, Sigma Aldrich, St. Louis, MO, USA) or YAP (1:100, #14074, Cell Signaling, Danvers, MA, USA), respectively, were added and incubated overnight at 4 °C. Afterwards sections were incubated with the respective secondary antibody 1:200 (goat anti-mouse Alexa Fluor 647, Thermo Fisher Scientific, Waltham, MA, USA) or 1:800 (goat anti-rabbit Alexa Fluor 555, Thermo Fisher Scientific, Waltham, MA, USA) in PBS containing 0.5 % Triton X-100. In the final step, nuclei were stained with DAPI (1:10,000 in PBS, Sigma Aldrich, St. Louis, MO, USA), and sections were mounted on coverslips with Aqua Polymount (Polysciences, Warrington, PA, USA). An Olympus IX83 inverted microscope (Olympus, Tokyo, Japan) was used to detect the immunofluorescent staining. FHL2 overall and nuclear staining on CIB- and MG-sections was described quantitatively as “none”, “weak”, “moderate” or “strong”. Therefore, at least 30 cells were counted on three independent sections per condition.

### 2.8 FUCCI cell cycle quantification

For cell cycle quantification in the biomaterials, Mel Im or MV3 labelled with the fluorescence-based ubiquitination cell cycle indicator (FUCCI) were used [8,9,14]. The FUCCI system was stably integrated into Mel Im and MV3 before, via lentiviral transduction of plasmid pBOB-EF1-FastFUCCI-Puro, as described elsewhere [15]. Once transduced, expression of fluorescence coupled portions of the cell cycle proteins Chromatin licensing and DNA replication factor 1 (Cdt1) (red) or geminin (green) indicate either G1 or S/G2/M state of the cells, respectively. During the transition from G1 to S phase, both proteins are present and merge producing a yellow fluorescence signal.

Quantification of the cell cycle reporter was performed as described previously [8]. Briefly, in three independent experiments, on days 1, 4 and 7 after printing, three merged z-stacks of the cells in the materials were taken in three different constructs in brightfield, CY3 and GFP channels using the Olympus IX83 fluorescence microscope and the cellSens software (Olympus, Tokyo, Japan), so in total 3 × 9 z-stacks were counted per condition. For quantification, maximum projections of the respective z-stacks were imported into the “Cell Counter” plugin of Fiji ImageJ software and cells were counted as red (G1), green (S/G2/M), or yellow (G1/S).

### 2.9 Plasmids

The luciferase reporter plasmid 3xAP1pGL3 (3xAP-1 in pGL3-basic) was a gift from Alexander Dent (Addgene plasmid # 40342; http://n2t.net/addgene:40342; RRID:Addgene_40342) [16]. The pGL4.23MCAT-LUC containing four TEAD-responsive elements and the respective mutated negative control plasmid pGL4.23MCATmut-LUC were a kind gift from Thomas Brabletz [17].

The AP-1/TEAD promotor co-occupation reporter plasmid was generated using the pGL3Promotor backbone from Promega (Promega Corp., Madison, WI, USA) and an amplicon of the promotor region of the *LIM domain containing preferred translocation partner in lipoma* (LPP) gene amplified by PCR from human cells using a primer pair containing *Sac*I restriction sites. Mutations in the TEAD or AP-1 binding regions were introduced using site-directed mutagenesis with mutated primer pairs deleting AP-1 or MCAT sites, respectively (Suppl. Table 2). A double mutant was generated by additionally mutating the TEAD binding site of the AP-1 mutant plasmid.

### 2.10 Luciferase assay

The activity of transcription factors was determined using luciferase (LUC) assays as described previously [6].

Mel Im or MV3 were transiently transfected with firefly-luciferase coding plasmid DNA containing specific transcription factor-responsive elements or the respective control vector (empty or mutated) and were co-transfected with a pRL-TK renilla luciferase control vector (Promega Corp., Madison, WI, USA) using Lipofectamine LTX/Plus reagent (Thermo Fisher Scientific, Waltham, MA, USA) according to the manufacturer's instructions. Cells were harvested 24 h after transfection and printed in CIB or MG. After another incubation for 48 h, the constructs were lysed and the firefly and renilla LUC activities were quantified by a luminometric assay (Promega Corp., Madison, WI, USA). The data were normalized to renilla LUC activity.

### 2.11 RNA sequencing

RNA from bioprinted constructs was extracted after two days in culture using Trizol® Reagent as described above. All of the RNA samples were examined for integrity and purity by the TapeStation 4200 (Agilent, Santa Clara, CA, USA). Library preparation was performed with three biological replicates using the TruSeq® Stranded Total RNA Library Prep Human/Mouse/Rat Kit according to the manufacturer's instructions (20020596, Illumina Inc., San Diego, CA, USA). The resulting libraries were checked for size (200–500 bp) by TapeStation 4200 (Agilent, Santa Clara, CA, USA) using the High-Sensitivity DNA Kit (Agilent, Santa Clara, CA, USA) and concentration by the Qubit 4 Fluorometer (Thermo Fisher Scientific, Waltham, MA, USA). Sequencing was performed on a HiSeq4000 Sequencing System with a paired-end module (Illumina, San Diego, CA, USA) according to the manufacturer's instructions. The samples were sequenced from each side of a fragment approximately 75 bp long with an average number of 20 million reads per sample. After quality check using FastQC [18] paired-end reads were aligned to the human reference genome (GRCh38.p5, release 24) using the STAR alignment software (v 2.7.9a) [19]. After mapping, only reads that mapped to a single unique location were considered for further analysis. The mapped reads were then used to generate a count table using the featureCounts software (Version 2.0.1) [20]. The raw reads were filtered, normalized and visualized using R (Version 4.3.2, The R Foundation for Statistical Computing, Vienna, Austria) [21]. The DESeq2 package (Version 1.42.1) was used for regularized logarithmic transformation of the data and for data exploration [22]. Differential expression analysis was performed using the DESeq2 standard approach. Adjusted p-values were calculated using the Benjamini-Hochberg method within DESeq2. Gene annotations were added to the results files using Ensemble data. Differentially expressed genes (DEGs) with an adjusted p-value <0.1 were regarded as statistically significant. Enrichment analyzes were done for overrepresentation analysis with Metascape v 3.5.20240901 [23] and for functional gene set enrichment with GSEA using MSigDB v 7.4 as previously described [9,[24], [25], [26]]. Enrichment maps were generated with cytoscape and enrichment map app [27,28]. Enriched MSigDB gene sets were considered significantly enriched with FDR <0.25. Annotation of AP-1 and E2F target genes inside the enrichment map was calculated with a Hyper-geometric test with a cutoff of 0.1.

### 2.12 Chromatin immunoprecipitation sequencing (ChIP-Seq) and data analysis

ChIP-Seq experiments with various melanoma cell lines (Sbcl-2, WM3211, WM1366, WM793, WM1158, WM9) were conducted previously (Bioproject: PRJNA1197724) and now bioinformatically reanalyzed [29]***.*** Shortly***,*** after ChIP with c-JUN (sc-1694, Santa Cruz, Dallas, TX, USA) and H3K27ac (ab4729, Abcam, Cambridge, UK) antibodies, respectively, the ChIP**-**DNA (10–50 ng) was adapter-ligated and polymerase chain reaction amplified according to the manufacturer's instructions (Illumina, San Diego, CA, USA). ChIP fragments were sequenced for 50 cycles on Illumina Genome Analyzer according to the manufacturer's protocol. Sequence tags of all experiments were mapped to the current human reference sequence (GRCh38/hg38) using Bowtie 2 (v 2.2.7) [30], and only uniquely mapped tags were used for downstream analysis. The identification of ChIP-Seq peaks was performed using a custom approach (HOMER: http://homer.ucsd.edu/homer/) that combines features of previously published methods [31,32]. Peaks were defined at a 0.001 estimated false discovery rate. Genome Ontology annotation and ChIP-Seq tag annotation of peak sets was performed using scripts provided by HOMER (based on GENCODE V24).

### 2.13 Statistics

Analysis and visualization of experimental results was done using GraphPad Prism 5 software (GraphPad Software Inc., San Diego, CA, USA). If not indicated otherwise, data were reproduced in 3 individual experiments and are shown as mean ± SEM. Comparisons between two groups were conducted using Student's unpaired *t*-test by default. FHL2 and TCF4 mRNA expression in CIB and MG were tested using the Wilcoxon matched-pairs signed rank test (paired, one-tailed). To test H3K23ac coverage quantification for significance a Wilcoxon test (paired, two-sided) was performed. For comparison of more than 2 groups we used one-way ANOVA followed by Tukey's Honest Significant Difference (HSD) post-test. In order to evaluate differences between groups on two independent variables, including the cell cycle states at different time points, the significance of immunostaining intensities or knockdown analysis, we used two-way ANOVA with Bonferroni post-test, respectively. A critical value of ∗*p* < 0.05 was considered statistically significant.

## 3 Results

### 3.1 RNA-seq data show an upregulation of genes associated with cell adhesion and motility in Cellink Bioink 3D culture

**Fig. 1:**
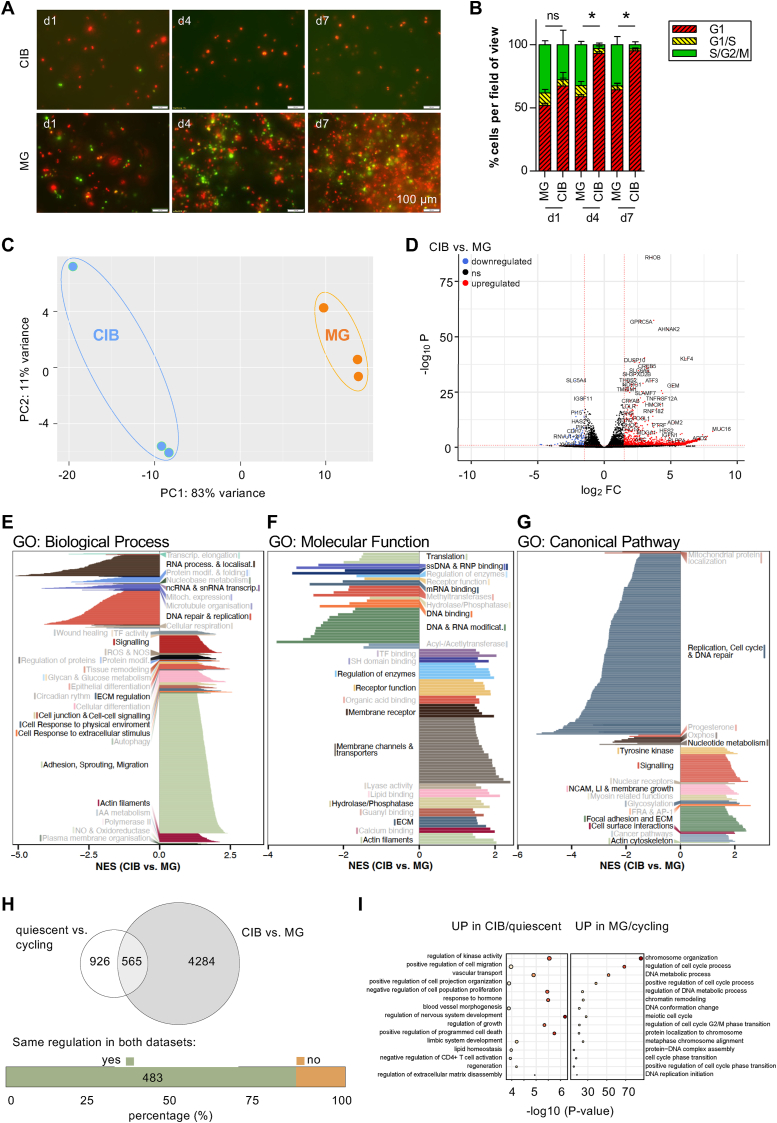
**Comparison of melanoma cell lines cultured in Cellink Bioink (CIB) or Matrigel (MG). A)** Microscopic images of MV3 FUCCI cultured in CIB or MG, respectively, on day 1, 4 and 7 after printing. Scale bar = 100 μm. **B)** Quantification of the FUCCI cell cycle indicator in MV3 cultured in CIB or Matrigel, respectively, on day 1,4 and 7 after printing shows the accumulation of cells in G1 phase in CIB. *∗p < 0.05 (Two-Way-ANOVA followed by Bonferroni post-test)*. **C)** Principal Component Analysis (PCA) of Mel Im RNA-Seq samples. **D)** Volcano plot of differentially expressed genes (DEGs) in CIB- compared to MG-cultured Mel Im cells. 2059 genes are significantly downregulated in CIB (log-fold change (LFC) < 0; p_adj_ < 0.1). Genes with a LFC <1.5 are colored in blue. 2790 genes are significantly upregulated in CIB (LFC >0; p_adj_ < 0.1). Genes with a LFC >1.5 are colored in red. Gene Set Enrichment Analysis (GSEA) of DEGs in CIB versus MG using the gene sets of **E)** GO: *Biological Process***F)** GO: *Molecular Function***G)** GO: *Cellular Component*. The enrichments are illustrated by either negative (downregulated in CIB vs. MG) or positive (upregulated in CIB vs. MG) normalized enrichment scores (NES) and the enriched gene sets were summarized by function. Each bar describes a gene set and the color describes the functional affiliation of the gene sets. **H)** The DEGs resulting from RNA-Seq of quiescent or cycling melanoma cells by La et al. and the DEGs comparing Mel Im cultivated in CIB- or MG show an overlap of 565 genes [33]. 483 genes (∼85 %) of the common DEGs show the same direction of regulation. **I)** Overrepresentation analysis with Metascape v 3.5.20240901 [23] of the common upregulated DEGs in CIB/quiescent and MG/cycling. The sizes of the dots indicate the gene set size and the color represents the statistical significance of the overrepresentation illustrated on the x-axis [-log10(P-value)].

Melanoma cells were shown to develop distinct cellular phenotypes when cultured 3D in CIB or MG [3]. The fluorescence-based ubiquitination cell cycle indicator (FUCCI) clearly revealed that the melanoma cell lines Mel Im (published in Ref. [6]) and MV3 cultured in CIB remained in G1-arrest, whereas those cultured in MG showed an ongoing cell cycle over up to 7 days (Fig. 1A). In line with previous results, after 7 days, 97.5 ± 1.1 % of MV3 cells cultivated in CIB were arrested in G1-phase, while only 63.0 ± 9.2 % cells cultivated in MG were in G1-and 32.4 ± 11.2 % in S/G2/M-phase, indicating active cycling of the cells (Fig. 1B). In order to define the molecular basis of these observed phenotypes, especially of the stop in proliferation, we performed RNA-Seq of the melanoma cell line Mel Im, which we had already used in previous studies, in the two different settings.

We first analyzed the gene expression pattern of the melanoma cells cultivated in the two different 3D cell culture models CIB and MG by RNA-Seq. Principal component analysis (PCA) of log-transformed normalized counts of the RNA-Seq data clearly shows striking differences in the gene expression pattern between the cells cultured in the two 3D models (Fig. 1C). Differential expression analysis using DESeq2 resulted in 4849 differentially regulated genes (log-fold change (LFC) > 0 (up): 2790 (57.5 %), LFC <0 (down): 2059 (42.5 %), p_adj_ < 0.1) (Fig. 1D**; Suppl. Data: 1_DEGs_CIB-MG**). To further address the biological meaning of our data, we performed Gene Set Enrichment Analysis (GSEA) of the significantly regulated genes (threshold p_adj_ < 0.1) and the results of all gene ontology annotations, *Biological Process* (Fig. 1E), *Molecular Function* (Fig. 1F) and *Canonical Pathways* (Fig. 1G), are illustrated. After gene set clustering, the results clearly revealed a major enrichment of gene sets involved in *DNA repair & replication* as well as *RNA processing* and *RNA transport* in MG-cultured Mel Im cells.

In previous studies, we could already show the potential of melanoma cells to be motile in CIB culture [6,7]. In line with these observations, genes associated to the GO term *adhesion, sprouting and migration* as well as *actin filaments and signaling* were enriched in CIB-cultured Mel Im. In addition, clustering genes upregulated in CIB resulted in an enrichment of gene sets regarding *cellular differentiation, cell to cell signaling* and *response to extracellular and physical stimulus*. These signatures are confirmed in GSEA results of GO molecular functions (Fig. 1F) and canonical pathways (Fig. 1G).

In summary, the GSEA findings are supporting active cell cycle progression in Mel Im cultured in MG and an intensive and dynamic interaction of cells with the microenvironment under CIB culture conditions. These interesting observations were further supported by *Canonical pathway* analysis, which revealed a strong correlation of downregulated genes in CIB to *Replication*, *cell cycle*, *and* *DNA repair* and of upregulated genes in CIB with *Signaling* and *ECM and focal adhesions* (just to name a few) (**Suppl. Data: 2_GSEA_BP, 3_GSEA_MF, 4_GSEA_CP)**.

To our knowledge, not many RNA-Seq datasets comparing quiescent/dormant and proliferative melanoma cells are publicly available. La et al. published a dataset comparing dormant Ki67^low^/p27^high^ and cycling Ki67^high^/p27^low^ populations of Mel-RM melanoma cells (GSE174520) [33]. Thus, we performed differential expression analysis using the respective publicly available RNA-Seq data of cycling and quiescent melanoma cells (**Suppl. Data: 5_DEGs_Cycling-Quiescent)** and merged the identified DEGs from their study with those of ours. Interestingly, we observed an overlap of 483 significant DEGs with matching regulation, being roughly half of the DEGs La et al. observed in total (Fig. 1H). These common DEGs were further analyzed using Metascape [23] to elucidate the potential cellular context of the respective genes. DEGs upregulated in CIB-cultured cells and quiescent/dormant melanoma cells are predominantly belonging to processes of *cell migration* as well as *cellular development*, and *ECM disassembly*. In contrast, in MG-cultured cells and cycling melanoma cells the upregulated genes are mainly involved in *chromosome organization, cell cycle*, and *DNA replication* (Fig. 1I, **Suppl. Data: 6_Metascape_CIB_up, 7_Metascape_CIB_down)**.

Taken together, our data and the comparison with the data from La et al. reveal that CIB culture is a highly suitable model to mimic melanoma quiescence/dormancy and even includes the microenvironmental factor cells usually lack in 2D culture.

### 3.2 Cell type classification by gene signatures of CIB and MG 3D cultured cells using scRNA-Seq data

**Fig. 2:**
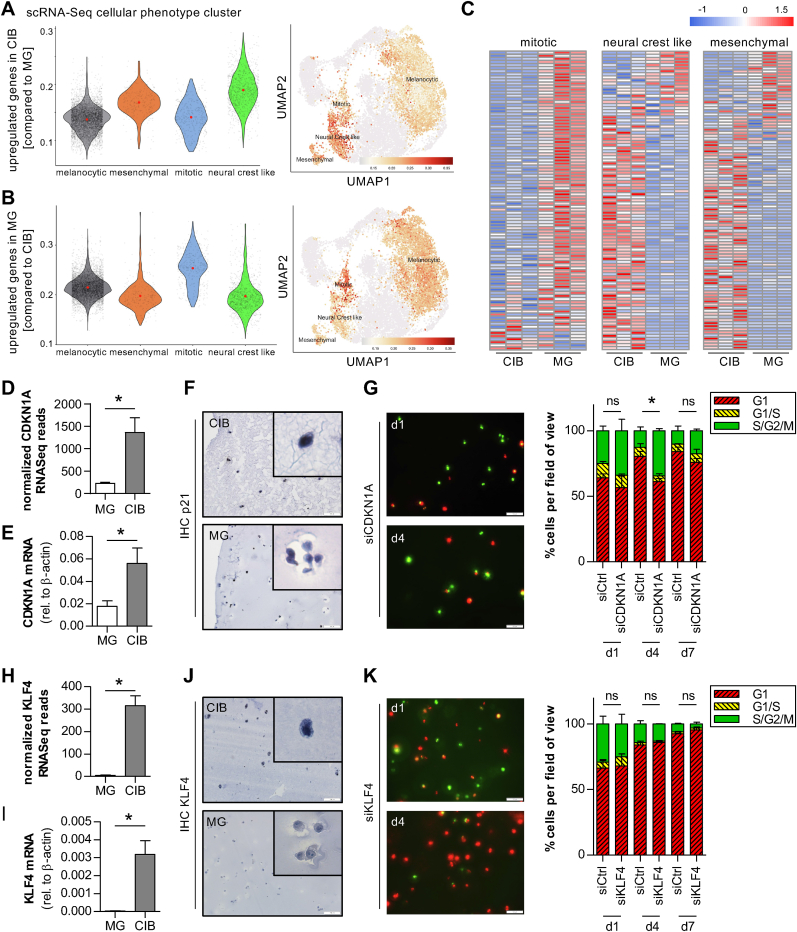
**CIB culture induces a stable mesenchymal phenotype of Mel Im.** Assignment of DEGs comparing CIB- and MG-cultured cells with published scRNA-Seq data of malignant melanoma [34]. The cell state annotated marker genes described by Pozniak et al. were used to match the CIB specific gene signature composed of upregulated genes (LFC >1.5) with the validated cell clusters in human melanoma tumor biopsy samples. **A)** Significantly upregulated genes in CIB cultivated cells showed an enrichment in the neural crest like and mesenchymal defined cell clusters and **B)** significantly upregulated genes under MG culture conditions illustrate an enrichment in the mitotic cell cluster. **C)** Expression status of the respective marker genes of the mitotic, neural crest like and mesenchymal cell type clusters [34] in CIB- and MG-cultured melanoma cells based on the scaled normalized RNA-Seq count data. **D)** CDKN1A RNA-Seq read counts (normalized to library size) in CIB- and MG-cultured Mel Im cells. **E)** CDKN1A (p21) mRNA expression analysis of Mel Im cultured in CIB or MG by qRT-PCR. **F)** Immunohistochemical staining for p21 on fixed and paraffin-embedded sections of CIB- or MG-cultured Mel Im. Scale bar = 100 μm. **G)** Representative images and quantification of Mel Im FUCCI after siPool mediated knockdown of p21 in CIB culture. Scale bar = 100 μm. **H)** KLF4 RNA-Seq read counts (normalized to library size) in CIB- and MG-cultured Mel Im cells. **I)** KLF4 mRNA expression analysis of Mel Im cultured in CIB or MG by qRT-PCR. **J)** Immunohistochemical staining for KLF4 on fixed and paraffin-embedded sections of CIB- or MG-cultured Mel Im. Scale bar = 100 μm. **K)** Representative images and quantification of Mel Im FUCCI after siPool mediated knockdown of KLF4 in CIB culture. Scale bar = 100 μm *∗p ≤ 0.05 (****D****,****E****,****H****,****I****: Student's t-test.****G****,****K****: Two-way ANOVA followed by Bonferroni post-test).*

As we were able to identify an induced phenotype switch by either CIB or MG culture conditions and the respective gene expression profile of the suggested dormant melanoma cells cultivated in CIB and the proliferative ones in MG, we further aimed to validate the discovered signatures on a single cell level. Pozniak et al. [34] published scRNA-Seq data of human melanoma metastatic biopsies, classifying cellular subtypes. These data clearly demonstrate melanoma heterogeneity and plasticity as they defined multiple (melanoma) cell type clusters by dimensionality reduction of the single-cell RNA-Seq data. Hence, Pozniak et al. defined melanocytic, neural-crest like, RNA-processing, antigen-presenting, stem-like, stress-like or mesenchymal cell types based on marker gene expression and thus the gene expression pattern of the melanoma subclusters (**Suppl. Data: 8_Pozniak *et al.* CellTypeCluster**). Based on this knowledge, we correlated the gene expression pattern of either CIB or MG cultivated melanoma cells to these subclusters to validate our assumption that CIB culture induces a quiescent/dormant and MG culture induces a proliferative phenotype. Therefore, we mapped the significantly upregulated genes in either CIB- or MG-cultured melanoma cells with the clusters melanocytic, mesenchymal, mitotic and neural-crest-like of the single-cell data by determining the gene set AUC scores using the AUCell package [35]. Indeed, the upregulated genes in CIB-cultivated cells clearly showed an enrichment in cell type clusters defined as mesenchymal and neural crest-like, whereas genes upregulated under MG conditions best matched the expression of mitotic cells (Fig. 2A and B). Additionally, overrepresentation analyses revealed a strong association of genes upregulated in CIB with mesenchymal and neural crest-like gene signatures, while upregulated genes in MG showed a significant enrichment within the melanocytic and mitotic subclusters defined by Pozinak et al. (Suppl. Fig. 1A). We further analyzed the expression status of the defined mitotic, mesenchymal or neural-crest-like gene signatures in our CIB and MG RNA-Seq data and could clearly validate the cell type clustering. The majority of genes annotated to the mitotic cell cluster are predominantly downregulated in CIB-cultured melanoma cells and those annotated to the neural crest-like and mesenchymal cell clusters upregulated compared to the gene expression of MG-cultured cells (Fig. 2C**, Suppl. Data: 9_DEGs_mitotic, 10_DEGs_mesenchymal, 11_DEGs_neural-crest-like**). To again validate these observations of a CIB-induced mesenchymal phenotype, we performed qRT-PCR for the mesenchymal marker *transcription factor 4* (TCF4) or the mitotic marker *lamin B1* (LMNB1) and saw up- or downregulation in CIB- versus MG-cultured cells, respectively (Suppl. Figs. 1B and C). These results clearly reveal that our newly identified gene expression profiles induced by the culture conditions CIB and MG reflect two different cell type clusters in malignant melanoma.

Thus, we further focused on identifying the molecular processes leading to the microenvironmentally induced phenotype switch. The mesenchymal to epithelial transition (MET) has been suggested to be a crucial process in the progression from the dormant to a proliferative cell state [36,37]. Hence, we aimed to classify the influence of this process in our setting by shifting the cellular phenotype of CIB-cultured cells back towards a more epithelial, proliferative state. Here, we focused on genes with a strong upregulation in CIB and a known impact on cell cycle progression or stemness. Our gene expression analysis clearly revealed elevated levels of p21, encoded by the cyclin dependent kinase inhibitor 1A gene (CDKN1A) (log2FC 2.52, p_adj_ 1.16E-21; Fig. 2D). P21 is known to bind to the cyclin E-CDK2 complex and thereby inhibits cell cycle transition from G1 to S. We experimentally confirmed the p21 upregulation in CIB-cultivated Mel Im cells by qRT-PCR and on protein level by IHC (Fig. 2E and F, Suppl. Fig. 2A). To validate the suggested direct effect of p21 on the observed dormant phenotype, we performed a knockdown of CDKN1A in the FUCCI-labelled melanoma cell lines Mel Im and MV3. Interestingly, culturing these CDKN1A-knockdown cells in CIB revealed a significantly decreased number of cells in G1 phase of the cell cycle compared to the control treatment after 4 days (Fig. 2G, Suppl. Fig. 2B). Still, there was no visible proliferation and after 7 days in total all cells had entered G1 cell cycle arrest again, indicating only insufficient p21 knockdown efficiency on cell proliferation in CIB.

These observations show that altered levels of p21 influence the characteristics of the dormant phenotype observed in CIB, however, further key players upstream of p21 must be involved and might be easier to target. Mining our RNA-Seq results, we observed KLF4, a potential activator of p21 by a specific Sp1-like-*cis*-element in the CDKN1A proximal promoter [38], to be highly upregulated in the dormant cells in CIB culture (log2FC 6.15, p_adj_ 1.05E-40, Fig. 2H, see also Suppl. Fig. 2C). The increased mRNA expression of the stemness marker KLF4 in CIB culture could be confirmed by qRT-PCR (Fig. 2I). Further, we analyzed the protein expression level by IHC and detected KLF4 exclusively in CIB but not in MG culture (Fig. 2J). However, after siRNA-mediated knockdown of KLF4, melanoma cells were still not able to strongly proliferate in CIB (Fig. 2K), indicating that KLF4 is not the key regulator of the dormant phenotype in CIB either. This classical approach of targeting cell cycle and stemness-associated genes was not sufficient to trigger proliferation of the cells in CIB culture.

Our results again point towards the importance of the ECM and indicate that mechanical cues from the matrix sensed by the cells might be the trigger of the signaling cascade inducing the quiescence we observe in CIB.

### 3.3 AP-1 and E2F are associated to gene signatures of cells cultivated in CIB or MG

**Fig. 3:**
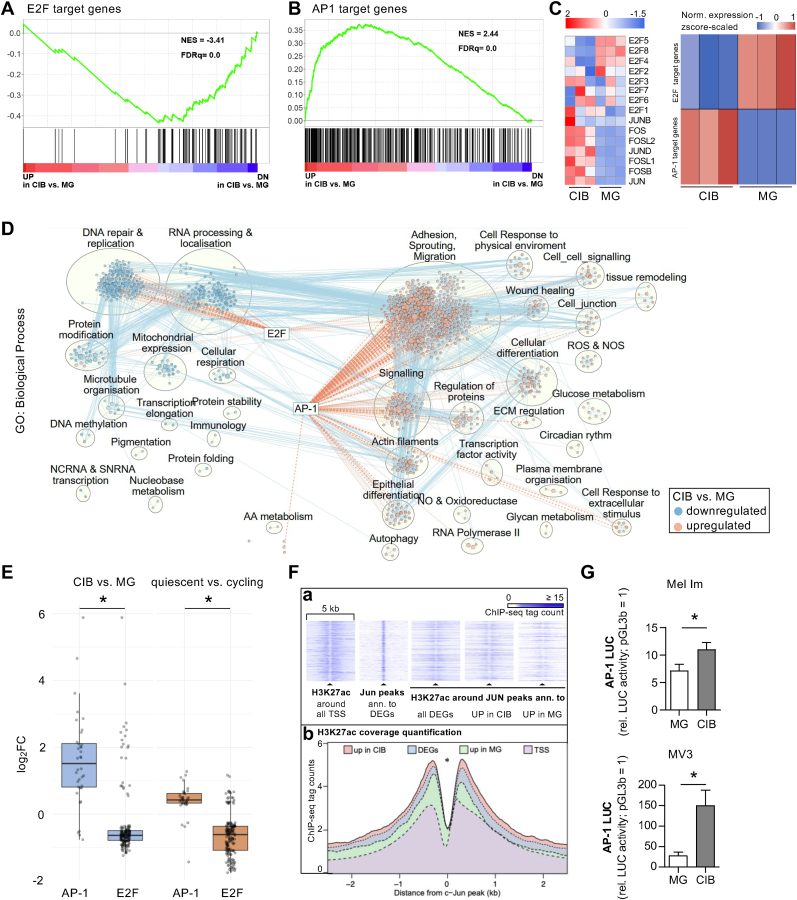
**Role of the transcription factors AP1 and E2F in the microenvironmentally induced phenotype switch.** Gene set enrichment analysis (GSEA) enrichment plots of **A)** E2F1 (TFTs) and **B)** AP-1 transcription factor targets among the differentially expressed genes in CIB compared to MG cultivated Mel Im. NES: normalized enrichment score; FDR: false discovery rate. **C)** Heatmap of selected E2F and AP-1 transcription factor family members, their expression status and the normalized expression scaled z-score of all predicted E2F and AP-1 target genes in CIB and MG culture conditions. **D)** RNA-Seq data network analysis of differentially expressed genes in CIB vs. MG culture by cytoscape utilizing the gene set GO: Biological Process. Networks with semantic annotations were summarized by AutoAnnotate and the presence of respective affected E2F or AP-1 target genes are illustrated by dotted lines. The blue coloring represents a downregulation and the red coloring an upregulation of the respective gene set cluster in CIB-cultured cells. **E)** Boxplot of potential E2F and AP-1 TFTs differentially expressed in CIB- vs. MG-cultured cells and quiescent vs dormant melanoma cells [33]. *∗p*_*adj*_*< 0.0001 (ANOVA test followed by Tukey's Honest Significant Difference (HSD) post-hoc test)* (CIB vs. MG: p_adj_ < 2E-16; Quiescent vs. Cycling: p_adj_ < 9.1E-10). **F) (a)** Heat maps and histograms of ChIP-Seq tag counts of the histone acetylation status (H3K27ac) in a 5-kb wide range around the TSS or JUN peaks, respectively. The annotated JUN peaks in transcriptionally active regions were assigned to all differentially expressed genes, to upregulated genes in CIB and to upregulated genes in MG-cultured Mel Im. TSS: transcription start site; ann.: annotated; DEGs: differentially regulated genes; UP: upregulated. **(b)** Quantification of **(a)***∗p < 2.2E -16 (Wilcoxon test, paired, two-sided)*. **G)** Luciferase assay of Mel Im or MV3 cultured in CIB or MG, respectively, shows increased activity of AP-1 in CIB culture. *∗p < 0.05 (Student's t-test).*

By comparing the gene expression pattern of CIB- and MG-cultured cells, we confirmed the CIB culture conditions as a suitable model for melanoma quiescence/dormancy. However, the underlying molecular mechanisms and key regulators inducing the observed phenotype switch stay elusive. Thus, we aimed to determine transcription factors, which might be involved in the environmentally induced changes of cellular behavior by performing target gene prediction analysis. We utilized GSEA on experimentally validated transcription factor target genes (MSigDB, C3: TFT) and revealed meaningful enrichments concerning the transcription factors E2F and AP-1. The transcription factor E2F is predicted to be a main regulator of downregulated DEGs (Fig. 3A, see also Suppl. Fig. 3A), whereas AP-1 family members seem to predominantly regulate genes, which are upregulated in CIB-cultured cells (Fig. 3B; see also Suppl. Fig. 3B). Supporting this, reduced expression of E2F factors like E2F5 (log_2_FC: −0.925; p_adj_: 0.103) or E2F8 (log_2_FC: −0.928; p_adj_: 0.0001) and induced expression of AP-1 factors like JUN (log_2_FC: 4.41; p_adj_: 5.58E-18) or FOSB (log_2_FC: 3.27; p_adj_: 0.003) could be observed in our RNA-Seq data. In addition, the predicted target genes of E2F and AP-1, respectively, showed a positive correlation between their expression status and the expression of the corresponding transcription factor (Fig. 3C). Interestingly, GSEA analysis of the respective deregulated E2F and AP-1 target genes clearly revealed an induction of genes annotated to functional clusters like *Adhesion*, *Sprouting and Migration*, as well as *Signaling and Cellular differentiation* in CIB-cultured cells. In contrast, gene sets related to proliferative processes like *DNA-repair & replication* and *RNA-processing & localization* are strongly downregulated in CIB culture (Fig. 3D). These findings could also be confirmed by analyzing the common DEGs from our dataset, comparing CIB and MG culture conditions, and the dataset from La et al. comparing classified quiescent and cycling cells (Fig. 3E**, Suppl. Data: 12_AP1_E2F targets CIB-MG, 13_AP1_E2F targets Cycl-Quies**).

As JUN showed the highest upregulation (log_2_FC: 4,41; p_adj_: 5.58E-18) amongst all upregulated AP-1 members in CIB-cultured cells (Fig. 3C), we speculated if this transcription factor might be the key driver of the observed phenotype switch. We aimed to determine the regulatory relevance of c-Jun on the identified upregulated genes in CIB-cultured melanoma cells and therefore reanalyzed our c-JUN ChIP-Seq data set conducted with 6 different melanoma cell lines (PRJNA1197724) [29]. Next, we combined the identified, annotated c-JUN ChIP-seq peaks and the DEGs in CIB compared to MG cultivated melanoma cells. Of the total of 117,793 c-JUN peaks, 7787 could be linked to upregulated DEGs in CIB and 4359 in MG cultivated cells. Interestingly, 1369 (of in total 2790) upregulated genes in CIB- compared to MG-cultured cells show a c-Jun peak in their respective promoter/enhancer region (Fig. 3Fa). Additional H3K27ac-ChIP-Seq experiments (PRJNA1197724) revealed histone acetylation around c-Jun peaks annotated to the identified upregulated genes under CIB culture conditions and thus confirmed that the corresponding regions are transcriptionally active. Comparison of H3K27ac histone modifications around either all identified differentially expressed genes or only up- or downregulated ones in CIB- versus MG-cultured cells clearly showed an increased acetylation status around c-Jun peaks of upregulated genes in CIB (Fig. 3Fb). Hence, a significant correlation of c-Jun-bound promoter/enhancer regions and the identified upregulated genes in CIB-cultured cells could be detected, supporting the regulatory importance of AP-1 inducing a dormant phenotype. To further validate this striking correlation between the expression of AP-1 transcription factors and upregulated genes in CIB-cultured cells, we performed AP-1-luciferase assays in the biofabricated constructs of melanoma cell lines in CIB or MG and revealed a significantly higher AP-1 activity in both Mel Im and MV3 in CIB culture compared to MG (Fig. 3G).

In summary, we could demonstrate a strong correlation of E2F or AP-1 activity in an either proliferative or dormant cellular phenotype, induced by the composition of the microenvironment.

### 3.4 Role of the mechanosensory transcription factor TEAD in the quiescent phenotype in CIB

**Fig. 4:**
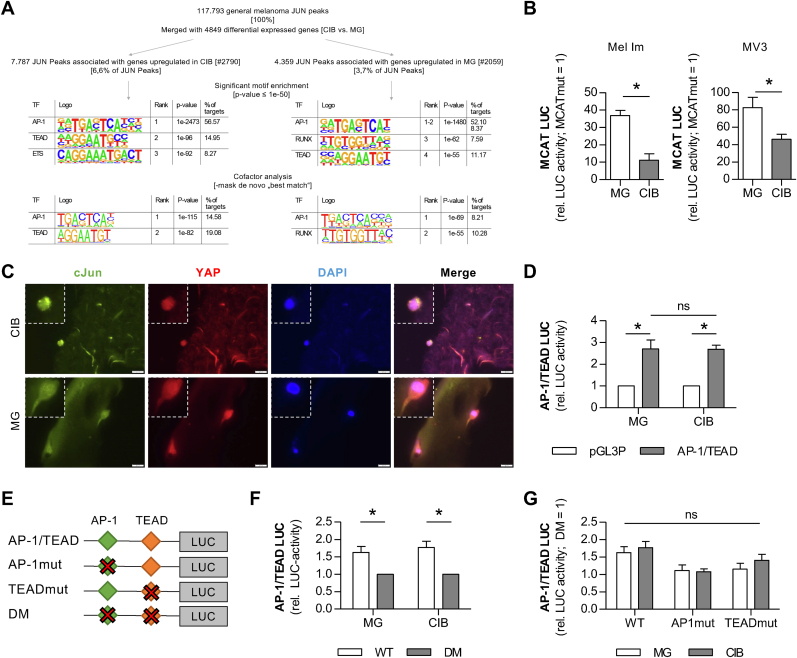
**Role of the mechanosensory TEAD transcription factor family in the quiescent phenotype induced in CIB. A)** Homer motif analysis in JUN-bound regions, which were derived from 5 different melanoma cell lines and associated with upregulated genes in CIB and MG, respectively. Motif enrichment was calculated with HOMER software, applying cumulative hypergeometric distribution adjusted for multiple testing with the Benjamini-Hochberg method. Potential cofactor binding was determined by masking the *de novo* best match. **B)** MCAT-Luciferase assays (reporter vector containing four MCAT-elements as binding sites for TEAD) of Mel Im (n = 3) or MV3 (n = 5) cultured in CIB or MG, respectively, show increased activity of TEADs in Matrigel. **C)** Immunofluorescent staining for c-Jun and the TEAD cofactor YAP on fixed and paraffin-embedded sections of CIB- or MG-cultured Mel Im. Scale bar = 20 μm. **D)** Luciferase assays with a plasmid carrying both an AP-1 and a TEAD binding site were conducted in Mel Im cultured in CIB or MG and revealed equal LUC-activity compared to the pGL3Promotor (pGL3P) empty vector in both conditions. **E)** Schematic overview of the binding-site (BS) mutants generated by side-directed mutagenesis of the AP-1/TEAD LUC-construct. AP-1/TEAD carries both intact BS, while in AP-1mut the AP-1, in the TEADmut the TEAD BS, and in the double mutant (DM) both BS were destroyed. **F)** AP-1/TEAD luciferase assays in Mel Im cultured in CIB or MG compared to the double mutant (DM). **G)** Luciferase assays comparing the normalized activity of AP-1/TEAD, AP-1mut, or TEADmut promotors in Mel Im cultured in CIB or MG. *∗p < 0.05 (****B****: Student's t-test,****D****,****F****,****G****: Two-Way-ANOVA followed by Bonferroni post-test).*

As we could identify the melanoma relevant transcription factor c-Jun as a main regulator of the microenvironmental induced phenotype switch by significantly changing the gene expression pattern, we further focused on potential mechanosensory cofactors involved in this regulatory process. Therefore, we again utilized the combination of the identified, annotated c-JUN ChIP-seq peaks and the DEGs in CIB- compared to MG-cultivated melanoma cells. We further characterized the identified c-JUN ChIP-Seq peaks associated to a potential upregulation of target genes in either CIB- or MG-cultivated cells by their motif composition. As expected, we could show a significant enrichment of the classical AP-1 binding site in a majority of the peaks (p-value = 1E-2473; ∼56 %). Other motifs that could be remodeled *de novo* in the c-JUN peaks are ETS-1, TEAD and RUNX sites. Further analysis of c-JUN co-associated TFs after masking the best match *de novo* motif AP-1 revealed the presence of TEAD motifs around c-JUN binding sites in regulatory regions of differentially upregulated genes in CIB, whereas those in MG showed co-associated RUNX motifs (Fig. 4A). It is already well known that the TEA domain (TEAD) transcription factor family plays a crucial role in cellular mechanosensing and thereby induced gene expression regulation. TEAD gets activated upon binding of its cofactors YAP or TAZ, however these factors showed no noticeable changes in their expression in CIB culture. Still, we were interested in the impact of the activity of mechanosensory TEADs in our setting as, interestingly, Liu et al. showed a co-occupation of AP-1 and TEAD in promotors of genes involved in migration and invasion in various cancer types [39]. TEAD factors contain a DNA-binding TEA-domain recognizing the consensus sequence (5′-CATTCC-3′) called MCAT element. To validate the potential association between c-Jun and TEAD in our setting, we performed a TEAD-responsive MCAT-element-based luciferase assay using Mel Im or MV3 from MG or CIB cultures and, surprisingly, we could detect significantly higher activity of TEAD in MG-cultured cells compared to CIB for both cell lines (Fig. 4B). An immunofluorescence co-staining of the AP-1 member c-JUN and the TEAD-cofactor YAP showed partial staining in the nucleus in Mel Im cultured either in CIB or in MG indicating equal activity of the respective factors (Fig. 4C).

To analyze a potential coactivity of the two transcription factors, we mined our dataset for genes upregulated in CIB with both an AP-1 and a TEAD binding site in their promotors and subsequently generated a LUC plasmid carrying the natural promotor of one of these genes, *LIM domain containing preferred translocation partner in lipoma* (LPP; log_2_FC: 1,05; p_adj_: 3,49E-13). In line with our previous results, we did not detect any significant differences in the luciferase assay measuring the activity of the promotor of LPP in CIB- or MG-cultured Mel Im (Fig. 4D). To define the individual impact of AP-1 or TEAD on the observed activity, we introduced mutations into the binding site of AP-1 (AP1mut) or TEAD (TEADmut) or both (double mutant (DM)) (Fig. 4E). While the AP-1/TEAD activity was significantly increased compared to the DM, the single mutations revealed only non-significant trends to slightly reduced activity (Fig. 4F and G). This hints towards the fact that both transcription factors bind and start transcription of the downstream gene, but that they function individually and do not need to cooperate for function.

Taken together, these results indicate, that TEAD and its targets are not decisive for the induction of the dormant phenotype in CIB, but that AP-1 functions either individually or together with other yet undefined transcriptional regulators.

### 3.5 Impairing mechanotransduction of melanoma cells breaks dormancy in CIB culture

**Fig. 5:**
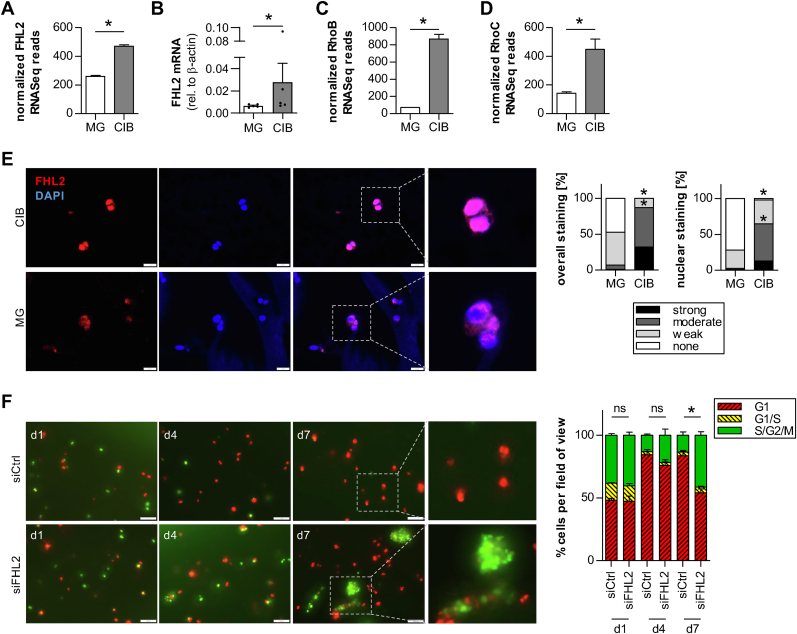
**Role of the mechanosensory gene FHL2 in the CIB-induced quiescent phenotype. A)** FHL2 RNA-Seq reads in Mel Im cultured in CIB or MG. **B)** FHL2 mRNA expression analysis of Mel Im cultured in CIB or MG by qRT-PCR; n = 5. **C)** RhoB RNA-Seq reads in Mel Im cultured in CIB or MG. **D)** RhoC RNA-Seq reads in Mel Im cultured in CIB or MG. **E)** Immunofluorescent staining for FHL2 on fixed and paraffin-embedded sections of CIB- or MG-cultured Mel Im reveals increased overall and nuclear staining of FHL2 in CIB. Scale bar = 20 μm. **F)** Representative images and quantification of Mel Im FUCCI after siPool mediated knockdown of FHL2 in CIB culture versus control. Outer right images show 2.5x magnifications of the marked sections from day 7. Scale bar = 100 μm *∗p < 0.05 (****A****,****C****,****D****: Student's t-test;****B****: Wilcoxon matched-pairs signed rank test;****E****,****F****: Two-Way-ANOVA followed by Bonferroni post-test).*

In the next step, we further investigated the involvement of potential mechanosensitive regulators interacting with AP-1 family members and highly upregulated genes in CIB-cultured melanoma cells to identify the molecular mechanism leading to the detected cell type change. Focal adhesions and the cytoskeleton are the first cellular sensors for mechanical cues coming from the ECM. Screening the upregulated genes in CIB-cultured cells in our dataset we identified the adaptor protein *four and a half LIM domains protein 2* (FHL2; log_2_FC: 0.86, p_adj_: 4.66E-06; Fig. 5A, see also Suppl. Fig. 2C), which is known to be able to shuttle and thereby transmit signals from focal adhesions in the membrane to the actin cytoskeleton and the nucleus [40]. qRT-PCR of Mel Im from CIB or MG culture also revealed a trend towards increased FHL2 mRNA expression in CIB (Fig. 5B). Additionally, we previously showed that FHL2 transmits signals of GTPases of the Rho family, which are also known to be involved in mechanotransduction, by this translocation [41,42]. Thus, we screened our sequencing data and found RhoB and RhoC to be significantly upregulated in the quiescent cells in CIB (Fig. 5C and D; RhoB: log_2_FC = 3.59, p_adj_ = 1.97E-86; RhoC: log_2_FC = 1.65, p_adj_ = 1.48E-10). It is further known that after translocation from the focal adhesions at the cell membrane into the nucleus, FHL2 stimulates the expression of its target genes, like for example p21 [43].

To determine FHL2 localization in 3D-cultured Mel Im, we performed immunofluorescence staining on CIB or MG sections and revealed not only increased overall staining of cells cultured in CIB, but also noticeably stronger nuclear staining compared to MG-cultured cells (Fig. 5E). Next, we established siPool-mediated FHL2 knockdown in Mel Im cultured in 2D on mRNA and protein-level and saw stable knockdown by up to 82 % (Suppl. Fig. 4). To validate its influence on the dormant phenotype in CIB, we performed FHL2 knockdown in Mel Im and seeded them into the dormancy-inducing hydrogel. Intriguingly, the FHL2 knockdown cells demonstrated the ability to strongly proliferate and form clusters during 7 days in CIB culture (Fig. 5F). As FHL2 has already been described as a transcriptional coactivator of AP-1 in murine fibroblasts [44], this goes in line with our observation of elevated AP-1 expression and activity in dormant cells in CIB. Thus, we were able to identify two main driver molecules, AP-1 and FHL-2, interacting with each other and linking microenvironmental changes and their sensing with transcriptional changes leading to a phenotype switch towards quiescence. However, further research is needed to elucidate the interplay of AP-1 and FHL-2 in the melanoma dormancy model.

## 4 Discussion

Commonly, tumor therapies are based on inhibition of cell growth and proliferation. However, metastasis, and, importantly, dormancy, survival and later progression to distant organs play a tremendous role in cancer and failure of cancer therapy. Models resembling these steps are rare but gain more and more attention.

We recently published a biofabrication process using the artificial hydrogel CIB (nanofibrillar cellulose + alginate), originally designed for cartilage tissue engineering, resulting in quiescence but survival of the melanoma cells embedded in this matrix, while they maintain a heterogeneous, predominantly cycling phenotype in most other hydrogels including alginate, MG or a mixture of alginate, hyaluronic acid and gelatin (Alg/HA/Gel) [3,[6], [7], [8],^45^]. Additionally, we know from a previous study, now further supported by our bioinformatical analyses, that CIB-cultured Mel Im also maintain their highly aggressive potential to invade into the blood system and settle at distant sites, well resembling the features of dormant cells *in vivo* [6,7]. Malignant melanoma is a highly heterogenic tumor with pronounced plasticity of the cells. Bittner et al. suggested early on that within this heterogeneity, specific transcriptional signatures might delineate melanoma cell subgroups [46]. In accord, Hoek et al. defined the so-called rheostat model of melanoma, revealing that melanoma cells are based on the expression level of the marker MITF either proliferative (MITF^high^) or migratory active (MITF^low^) [47]. In line with this definition, in a previous study, we could already show the MITF^low^ expression status of melanoma cells in CIB versus MG culture [6]. In recent works from Karras et al. and Pozniak et al., single-cell RNA-Seq of murine or human biopsies, respectively, was used to define and classify melanoma cells into multiple subtypes [34,48]. These include “mitotic” or “mesenchymal” phenotypes, also being characterized by an either proliferative and MITF^high^ or invasive and MITF^low^ phenotype, respectively [34]. Intriguingly, here we could show that based on a comparison of the gene clusters described by Pozniak and colleagues with the DEGs from our RNA-Seq data set, CIB-cultured cells can be classified as predominantly mesenchymal, whereas MG-cultured cells are mainly mitotic. Our observations are in line with observations of various groups showing a mesenchymal cell type of cancer stem cells, dormant cells or therapy resistant cells in breast cancer, squamous cell carcinoma or malignant melanoma, respectively [34,36,49]. These correlations emphasize the relevance and applicability of the CIB-culture to model tumor dormancy for the in-depth analysis of this yet unsolved threat in melanoma therapy.

The microenvironment, here resembled by different hydrogels, has a tremendous impact on gene expression and accordingly on the phenotype cells develop, yet it is unclear which features trigger and convey the dormant phenotype. Compared to MG, in CIB, melanoma cells face physical confinement and a rather stiff microenvironment [7]. Additionally, the cells cannot adhere to the alginate-cellulose matrix or digest it, as neither alginate nor cellulose offers recognition sites for integrins or MMPs, respectively. The finding that a hydrogel, in which no direct cell-matrix adhesion can be build, led to quiescence was astonishing as the same melanoma cells easily proliferate in soft agar colony formation assays [4]. We suggest that the interaction of cells with the dormancy-inducing hydrogel CIB is rather dependent on mechanical forces coming from the matrix. Typical mechanical cues are hydrostatic pressure, fluid shear stress, tensile force, extracellular matrix stiffness or elasticity, and extracellular fluid viscosity [50]. Cells can sense and react to mechanical stimuli via a range of mechanosensors and downstream signaling pathways. They do so by passing signals from the membrane or the cytoskeleton on to the nucleus, where gene expression patterns get adjusted. For tumor cells *in vivo* that means that changes in the extracellular matrix, e.g. after chemotherapy, through ageing or settling of cells in a new niche after dissemination, may lead to altered gene expression inducing invasion, dormancy, therapy resistance or ultimately metastasis [51].

Different groups also observed that matrix composition or physical confinement induce a dormant cell state of different tumor types. Preciado et al. inhibited proliferation of prostate, ovarian, and breast cancer cells through a stiff silica-PEG gel, in which cells entered quiescence [52]. Also Pradhan et al. were able to induce breast cancer cell quiescence, by embedding the cells in PEG-fibrinogen gels [53]. However, as recently reviewed, there are hardly *in vitro* models for the analysis of melanoma dormancy [54]. Yet, so far Liu et al. could show that stem-like, tumor-repopulating melanoma cells cultured in fibrin gels of different stiffness, develop a dormant phenotype only in the stiff gel, indicating that matrix mechanics play an important role in melanoma dormancy as well [55]. They suggest the induction of dormancy in their setting via an epigenetic program initiated by the translocation of the GTPase Cdc42 into the nucleus, leading to transcription of the enzyme Tet2 and finally to activation of p21 and p27.

In our present study, RNA sequencing of quiescent melanoma cells cultured in CIB and proliferating cells in MG, gives an insight to the immense scope of gene expression changes caused by the extracellular matrix only.

While the Tea-Domain (TEAD) transcription factor family is one famous transcription factor family known to be involved in mechano-signaling in cancer progression via the end effectors of the Hippo-signaling cascade YAP and TAZ [56,57], we did not reveal a major relevance for the observed quiescent phenotype in CIB culture. While Liu et al. showed a co-occupation of AP-1 and TEAD in promotors of genes involved in migration and invasion, we could not detect a cooperative effect on the transcription of genes in quiescent cells in CIB, but an impressive activity of AP-1 alone and loss of activity of E2F [39]. Tang et al. already showed for skeletal stem cells that in a model where stiff 3D ECM could not be remodeled and cells stayed in a small and rounded shape, YAP/TAZ were inhibited, which might also be the case in our melanoma setting [58].

In the physiological setting, matrix stiffening is a very important process in tumor development and progression, e.g. by increased matrix production of cancer associated fibroblasts (CAFs) leading to increased pressure in the tumor bulk [59]. The necessary mechanosensation of cells is said to be achieved by an interplay of the actin cytoskeleton and the adhesion complexes that physically connect the cytoskeleton to the ECM. Accordingly, the loss of cell matrix adhesion, e.g. upon dissemination of the cells and entering into the blood stream, is also a crucial stimulus, leading to altered molecular signaling [60]. For example, Wixler et al. showed that the transcriptional coactivator FHL2 localizes in focal adhesion complexes of cultured cells [40]. Müller et al. could reveal the translocation of FHL2 into the nucleus and subsequent activation of gene expression dependent on FHL2, induced by extracellular stimuli through Rho signaling [41]. While tumor cells are known to be able to grow and proliferate attachment-independently, our data indicate that as a result of the loss of cell-matrix adhesion in combination with the increased matrix stiffness in CIB, FHL2 usually localized at focal adhesion sites and in the cytoplasm, shifts into the nucleus, where it might contribute to the induction of cellular quiescence. Supporting this assumption, Nakazawa et al. suggest a force dependent regulation of p21 by FHL2 in melanoma [43]. On 2D substrates they found a nuclear FHL2 localization on soft and a membranous localization on stiff surfaces. In our 3D CIB culture, we observed the nuclear localization of FHL2 in the stiff CIB matrix, potentially due to the absence of focal adhesions, which are also rare on soft surfaces. In breast cancer cells an interaction of FHL2 and p21 was already shown by different groups. For example, Martin et al. also observed that downregulation of FHL2 abrogated cell-cycle dependent upregulation of p21, fitting our observations of increased p21 levels in CIB culture [61]. Interestingly, they found that downregulation of FHL2 led to decreased ability of MDA-MB-231 cells to form colonies in soft agar, while we found the opposite result in melanoma cells in the relatively stiff CIB matrix, again supporting an important role of the matrix stiffness and composition for FHL2 function. In line with our observations, Bakhshandeh et al. published a study on a dormancy-inducing alginate matrix for MDA-MB-231 and MCF7 cells only recently, where they also detected a mechanosensitive FHL2-p21 signaling axis being responsible for the cell cycle arrest under these growth restrictive conditions [62].

In our dataset, we found a significant upregulation of AP-1 factors and their target genes, hinting towards their regulation by FHL2. Intriguingly, Morlon et al. could define FHL2 as a transcriptional coactivator of AP-1 in murine fibroblasts and fibroblast-like cells [44].

Our data suggest that FHL2 is one of the first sensors mediating the stimulus from the microenvironment into the nucleus where it orchestrates the transcription of its targets including p21 and AP-1, thereby inducing the quiescent phenotype. Additionally, a study by Westphal et al. shows that an increased level of FHL2 leads to poor patient survival in melanoma. They associate their observation with increased metastatic potential of the cells, which is also reflected in the CIB setting [63].

While the model we present here offers great opportunities for dormancy-research focusing on the impact of the ECM, it also shares some limitations with existing models. First of all, the time factor that can be seen as an advantage on the one hand, might also be critical on the other, as dormant cells survive up to decades in patients, while under laboratory conditions we had maximum observation times of 14 days. We cannot estimate the scope of the changes happening under *in vivo* conditions during longer periods of time. Additionally, one has to keep in mind that stromal cells and the immune-microenvironment, which might have significant impact on the development and maintenance of dormancy *in vivo,* are missing here. However, bioprinting offers great opportunities to expand this fundamental model to a more complex system, including further cell types and vascularization and to investigate their respective impact on dormancy in a controlled and isolated manner.

Taken together, with this study we first performed an in-depth molecular characterization of Cellink Bioink (CIB) culture and suggest it as an attractive model for the impact of the extracellular matrix on dormant melanoma. We combined RNA-Seq, bioinformatical analysis and functional assays, in order to better understand the observed phenotypes, especially the dormancy of melanoma cells in CIB. Here, we were able to point out a mesenchymal phenotype of the dormant melanoma cells and additionally detected FHL2 as one major regulator of this dormant state. Further research is needed to determine the signaling pathways involved in detail, as well as its potential use as a novel therapeutic target to drive dormant cells back into a mitotic/proliferative state and to make them accessible for chemotherapy again.

We conclude, that this biofabricated model, in combination with the delivered dataset, can be used to further elucidate the mechanisms of dormancy and metastasis, in order to develop novel therapeutic options targeting dormant cells.

## CRediT authorship contribution statement

**Sonja K. Schmidt:** Writing – review & editing, Writing – original draft, Visualization, Validation, Supervision, Methodology, Investigation, Formal analysis, Data curation, Conceptualization. **Stefan Fischer:** Writing – review & editing, Writing – original draft, Visualization, Validation, Investigation, Formal analysis, Data curation. **Zubeir El Ahmad:** Writing – review & editing, Writing – original draft, Visualization, Methodology, Formal analysis, Data curation. **Rafael Schmid:** Writing – review & editing, Validation, Methodology, Investigation. **Eric Metzger:** Writing – review & editing, Validation, Resources, Methodology. **Roland Schüle:** Writing – review & editing, Validation, Resources, Methodology. **Claus Hellerbrand:** Writing – review & editing, Writing – original draft, Supervision, Methodology, Conceptualization. **Andreas Arkudas:** Writing – review & editing, Validation, Supervision, Methodology, Conceptualization. **Annika Kengelbach-Weigand:** Writing – review & editing, Validation, Supervision, Investigation, Formal analysis, Conceptualization. **Melanie Kappelmann-Fenzl:** Writing – review & editing, Writing – original draft, Visualization, Validation, Supervision, Methodology, Investigation, Formal analysis, Data curation, Conceptualization. **Anja K. Bosserhoff:** Writing – review & editing, Writing – original draft, Visualization, Supervision, Project administration, Methodology, Funding acquisition, Formal analysis, Data curation, Conceptualization.

## Statement of significance

Modeling tumor dormancy is difficult, as in animal models *in vivo* dormant tumor cells are hard to detect and to retain in a dormant state and 2D models lack the complexity of the microenvironment. We observed that tumor cells stay quiescent when 3D printed in the hydrogel *Cellink Bioink* (CIB). Here, we performed molecular characterization of these sleeping cells compared to proliferating cells in Matrigel (MG), using RNA-Seq with subsequent in-depth bioinformatical analyses, supported by 3D *in vitro* assays. Thereby, we were able to point out that quiescent cells in CIB share multiple characteristics and gene-regulation patterns with dormant or therapy resistant cells, undermining the model as a valid platform for the analysis of this phenomenon.

## Funding

The work was funded by grants from the 10.13039/501100001659Deutsche Forschungsgemeinschaft (DFG, German Research Foundation) to the TRR225 (project number 326998133 subproject C03, Z03) and to C.H. and A.B. (project numbers HE2458/20-1; BO1573).

## Declaration of competing interest

The authors declare no conflict of interest.

## Data Availability

The RNA-Sequencing data used in this study have been deposited in the NCBI BioProject database (https://www.ncbi.nlm.nih.gov/bioproject/) and can be accessed with the BioProject accession number PRJNA1197413. Other data will be made available on request.

## Appendix A Supplementary data

Multimedia component 1 1_**DEGs_CIB-MG**: Differential expression analysis results from RNA-Seq data comparing CIB versus MG cultivated melanoma cells (Mel Im). 2_**GSEA_BP**: Gene Set Enrichment Analysis (GSEA) results of DEGs in CIB versus MG using the gene set GO: Biological Process. Cluster annotation and direction of regulation are added in the last two columns. 3_**GSEA_MF**: Gene Set Enrichment Analysis (GSEA) results of DEGs in CIB versus MG using the gene set GO: Molecular Function. Cluster annotation and direction of regulation are added in the last two columns. 4_**GSEA_CP**: Gene Set Enrichment Analysis (GSEA) results of DEGs in CIB versus MG using the gene set GO: Cellular Component. Cluster annotation and direction of regulation are added in the last two columns. 5_**DEGs**_Cycling-Quiescent: Differential expression analysis results from public RNA-Seq data (La et al. [33]) comparing quiescent versus cycling melanoma cells. 6_**Metascape_CIB_up**: Metascape functional analysis results of common upregulated genes in CIB/quiescent melanoma cells. 7_**Metascape_CIB_down**: Metascape functional analysis results of common downregulated genes in CIB/quiescent melanoma cells. 8_**Pozniak_CellTypeCluster**: Gene Signatures of melanocytic, mitotic, neural crest like and mesenchymal melanoma cell type clusters (Pozniak et al. [34]). 9_**DEGs_mitotic_CIB-MG**: Overlap of differentially expressed genes in CIB versus MG culture with gene signature of mitotic melanoma cell type cluster (Pozniak et al. [34]). 10_**DEGs_mesenchymal_CIB-MG**: Overlap of differentially expressed genes in CIB versus MG culture with gene signature of mesenchymal melanoma cell type cluster (Pozniak et al. [34]). 11_**DEGs_neural-crest-like_CIB-MG**: Overlap of differentially expressed genes in CIB versus MG culture with gene signature of neural crest-like melanoma cell type cluster (Pozniak et al. [34]). 12_**AP1-E2F targets CIB-MG**: List of predicted AP1 and E2F target genes differentially expressed in CIB versus MG melanoma cells. 13_**AP1-E2F targets Quiescent-Cycling**: List of predicted AP1 and E2F target genes differentially expressed in quiescent versus cycling melanoma cells. Multimedia component 1

Supplementary Figure 1 **Overrepresentation analysis of upregulated genes within melanoma signature genes and expression of marker genes.** Supplementary Fig. 2: **Analysis of the expression of p21 and its regulators.** Supplementary Fig. 3: **E2F and AP1 gene and target gene regulation.** Supplementary Fig. 4: **Establishment of the FHL2 knockdown in Mel Im.** Supplementary Table 1: Oligonucleotides used for quantitative PCR. Supplementary Table 2: Oligonucleotides used for cloning and site-directed mutagenesis. Supplementary Figure 1
