# Supplementary material for: Modeling a mesenchymal cell state by bioprinting for the molecular analysis of dormancy in melanoma
Source: Mater Today Bio. 2025 Mar 18;32:101674. doi: 10.1016/j.mtbio.2025.101674 (PMC11979991; doi:10.1016/j.mtbio.2025.101674)
Supplement: Supplementary Figure 1 — Overrepresentation analysis of upregulated genes within melanoma signature genes and expression of marker genes. Supplementary Fig. 2: Analysis of the expression of p21 and its regulators. Supplementary Fig. 3: E2F and AP1 gene and target gene regulation. Supplementary Fig. 4: Establishment of the FHL2 knockdown in Mel Im. Supplementary Table 1: Oligonucleotides used for quantitative PCR. Supplementary Table 2: Oligonucleotides used for cloning and site-directed mutagenesis. [file mmc2.pdf]

Supplementary Figure 1

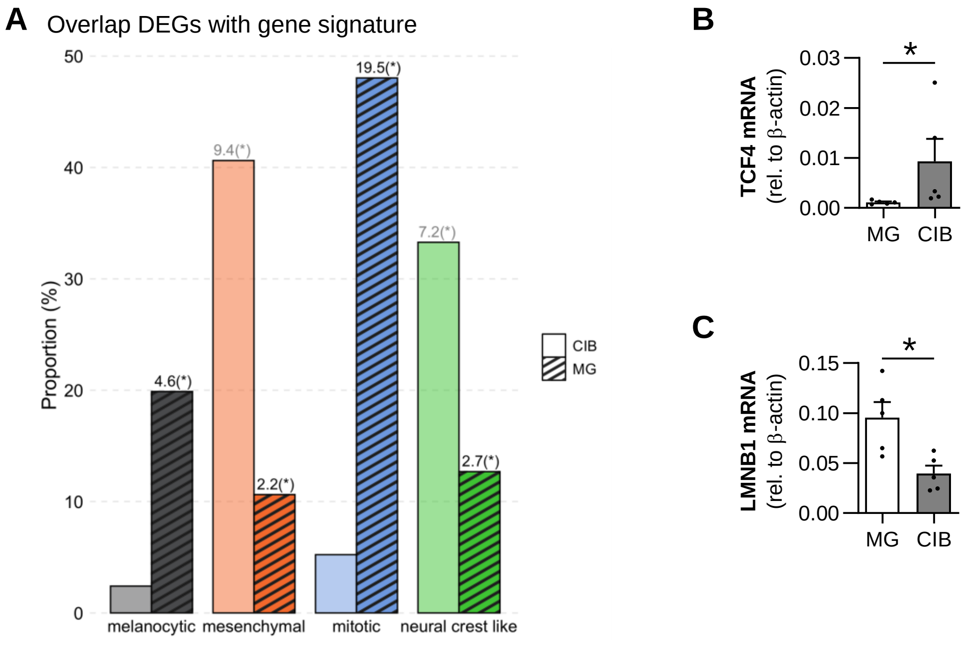

Supplementary Figure 1: Overrepresentation analysis of upregulated genes within melanoma signature genes and expression of marker genes. A) Barplot depicting the proportion of overlaps between up-regulated genes in either CIB (transparent/light) or MG (opaque/dark) with genes assigned to the different melanoma signature clusters by Pozniak et al. [34]. For statistically significant results *(*p-value < 0.01: Fisher's exact test)*, the odds ratio is given above individual bars, representing the strength of the association between gene upregulation and membership in the gene signature. **B)** TCF4 mRNA expression analysis of Mel Im cultured in CIB or MG by qRT-PCR, n = 5, *p < 0.05 (Wilcoxon matched-pairs signed rank test (paired, one-tailed)). **C)** LMNB1 mRNA expression analysis of Mel Im cultured in CIB or MG by qRT-PCR, n = 5, **p < 0.05 (Student's t-test).*

Supplementary Figure 2

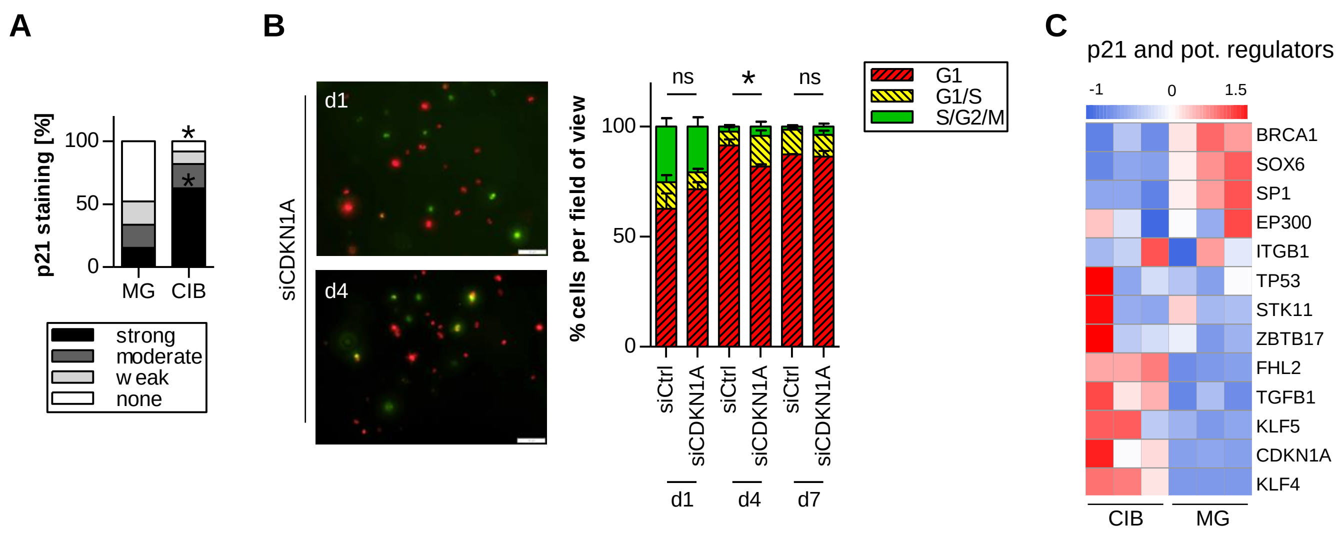

Supplementary Figure 2: Analysis of the expression of p21 and its regulators. A) Quantification of p21 staining intensity in MG versus CIB cultured Mel Im. **p < 0.05 (Two-way ANOVA followed by Bonferroni post-test).* **B)** Representative images and quantification of MV3 FUCCI after siPool mediated knockdown of p21 in CIB culture. Scale bar = 100 µm **p < 0.05 (Two-way ANOVA followed by Bonferroni post-test).* **C)** Expression status of CDKN1A (p21) and its potential regulators in CIB- and MG-cultured melanoma cells based on the scaled normalized RNA-Seq count data.

Supplementary Figure 3

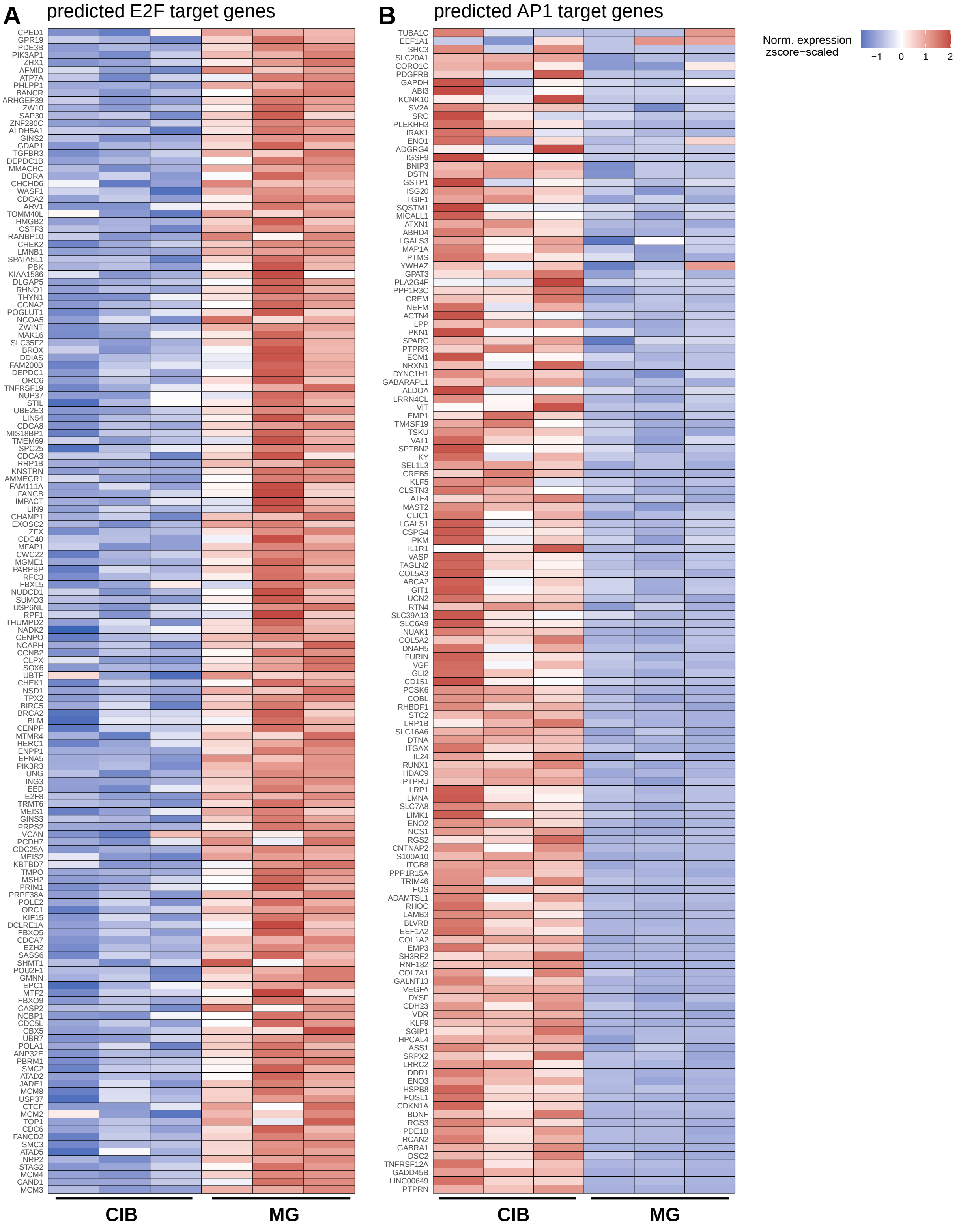

Supplementary Figure 3: E2F and AP1 gene and target gene regulation. Heatmap of potential **A)** E2F and **B)** AP-1 target genes from MSigDB (C3: TFT) differentially expressed in CIB compared to MG cultivated Mel Im cells.

Supplementary Figure 4

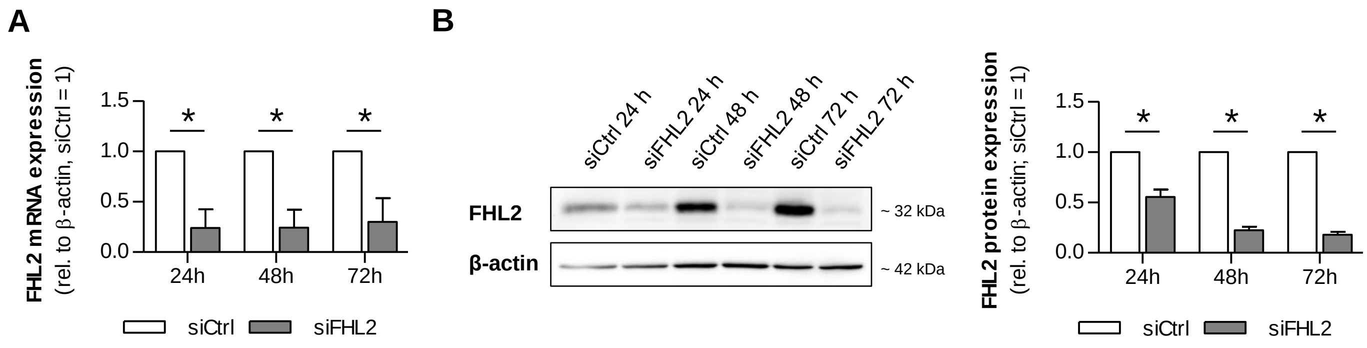

Supplementary Figure 4: Establishment of the FHL2 knockdown in Mel Im. FHL2 knockdown validation on **A)** mRNA-level by qRT-PCR and **B)** protein level by Western Blot and densitometric evaluation. **p < 0.05 (Two-way ANOVA followed by Bonferroni post-test).*

Supplementary Table 1

Supplementary Table 1: Oligonucleotides used for quantitative PCR.

Gene	5'-3' sequence forward	5'-3' sequence reverse
hACTB	CTACGTCGCCCTGGACTTCGAGC	GATGGAGCCGCCGATCCACACGG
hCDKN1A (p21)	CGAGGCACCGAGGCACTCAGAGG	CCTGCCTCCTCCCAACTCATCCC
hFHL2 (SLIM3)	CAGTGCAAAAAGCCCATCAC	GTTATGCCACTGCCGTTCCT
hKLF4	CCCTGGGTCTTGAGGAAGTG	GGCATGAGCTCTTGGTAATGG
hLMNB1	TATGAGTACAAGCTGGCGCA	TCTCATGCGGCTTTCCATCA
hTCF4	CAAGAGGCAAGATGGAGGGC	GTAATGTGTGCTGCCGGACT

Supplementary Table 2

Supplementary Table 2: Oligonucleotides used for cloning and site-directed mutagenesis.

Primer name	5'-3' sequence forward	5'-3' sequence reverse
LPPKLuc_TEAD_AP1_SacI	GACGAGCTCAGCCTTTCAGACCAGTCACTTC	GACGAGCTCCAGGCCCTCAGTCCACTACG
AP1_mut	[Phos]AGAGGGAGGTCACGCAGGGT	[Phos]CTTCCAATAGATCAGCCTGG
TEAD_mut	[Phos]CCCCTTCCGCACTCTGTCCCTT	[Phos]GAAGGGTGGGGGCAGGGTGG
